# Comparison of Phenylephrine and Ephedrine in Treatment of Spinal-Induced Hypotension in High-Risk Pregnancies: A Narrative Review

**DOI:** 10.3389/fmed.2017.00002

**Published:** 2017-01-20

**Authors:** Sasima Dusitkasem, Blair H. Herndon, Monsicha Somjit, David L. Stahl, Emily Bitticker, John C. Coffman

**Affiliations:** ^1^Department of Anesthesiology, The Ohio State University Wexner Medical Center, Columbus, OH, USA; ^2^Ramathibodi Hospital, Mahidol University, Bangkok, Thailand; ^3^Srinagarin Hospital, Khonkaen University, Khon Kaen, Thailand; ^4^University of Cincinnati College of Medicine, Cincinnati, OH, USA

**Keywords:** phenylephrine, ephedrine, hypotension, preeclampsia, uteroplacental insufficiency, fetal compromise

## Abstract

**Purpose:**

To compare maternal and fetal effects of intravenous phenylephrine and ephedrine administration during spinal anesthesia for cesarean delivery in high-risk pregnancies.

**Source:**

An extensive literature search was conducted using the US National Library of Medicine, MEDLINE search engine, Cochrane review, and Google Scholar using search terms “ephedrine and phenylephrine,” “preterm and term and spinal hypotension,” “preeclampsia and healthy parturients,” or “multiple and singleton gestation and vasopressor.” Society of Obstetric Anesthesia and Perinatology meeting abstracts for the past 4 years were also searched for relevant studies.

**Principle findings:**

Both phenylephrine and ephedrine can be safely used to counteract hypotension after spinal anesthesia in patients with uteroplacental insufficiency, pregnancy-induced hypertension, and in non-elective cesarean deliveries. Vasopressor requirements before delivery in high-risk cesarean sections are reduced compared to healthy parturients. Among the articles reviewed, there were no statistically significant differences in umbilical arterial pH, umbilical venous pH, incidence of fetal acidosis, Apgar scores, or maternal hypotension when comparing maternal phenylephrine and ephedrine use.

**Conclusion:**

From the limited existing data, phenylephrine and ephedrine are both appropriate selections for treating or preventing hypotension induced by neuraxial blockade in high-risk pregnancies. There is no clear evidence that either medication is more effective at maintaining maternal blood pressure or has a superior safety profile in this setting. Further investigations are required to determine the efficacy, ideal dosing regimens, and overall safety of phenylephrine and ephedrine administration in high-risk obstetric patients, especially in the presence uteroplacental insufficiency.

## Introduction

Maintenance of hemodynamic stability from a sympathetic blockade after neuraxial anesthetic techniques for cesarean delivery remains a significant clinical problem (1). To counteract maternal hypotension, intravenous fluid and vasopressor drugs are required.

Historically, ephedrine was considered the preferred vasopressor for management of spinal-induced hypotension in healthy parturients. Ephedrine has a relatively slow onset and long duration of action compared to phenylephrine and has a predominantly β-agonist effect (2). Studies in pregnant ewes demonstrated that ephedrine was effective in maintaining arterial blood pressure and was associated with greater preservation of uteroplacental blood flow compared with other vasopressors (3, 4). Historically, phenylephrine, a direct α1-agonist, was avoided due to concerns regarding potential uterine blood flow reduction (3). However, more recent clinical evidence has consistently demonstrated that phenylephrine is effective for maintaining blood pressure during elective cesarean deliveries with spinal anesthesia, does not exert an adverse effect on the fetus and is associated with a lower rate of fetal acidosis compared to ephedrine (5, 6).

In 2002, a quantitative systemic review by Lee et al. examined the role of ephedrine and phenylephrine in obstetric patients. The authors reported that phenylephrine use was associated with higher umbilical arterial (UA) pH values compared to ephedrine (2). Subsequent studies conducted in healthy parturient undergoing elective cesarean deliveries have consistently demonstrated that phenylephrine use reduces incidence of fetal acidosis compared to ephedrine (5–9) and is more effective at maintaining maternal blood pressure (7, 8) and preventing intraoperative nausea and vomiting (IONV) (5, 7, 8) compared to ephedrine. It has been demonstrated that ephedrine crosses the placenta to a greater extent than phenylephrine and stimulation of β-adrenergic receptors in the fetus results in an increased fetal metabolic rate (5, 7, 8). Ephedrine-induced fetal tachycardia and acidosis appears to depend on dosage and timing of drug administration prior to delivery (8–10).

There is a paucity of evidence to guide clinical decisions regarding vasopressor use in non-elective cesarean deliveries or high-risk parturients. The majority of data that have shaped current practices of vasopressor use for spinal-induced hypotension have been done in healthy women undergoing elective cesarean deliveries. These results cannot necessarily be extrapolated to patients diagnosed with impaired uteroplacental blood flow or with pregnancy-induced hypertension. This review examines the available evidence regarding the efficacy and safety of phenylephrine and ephedrine administration in high-risk cesarean deliveries.

## Methods

In order to compare maternal and fetal effects of intravenous phenylephrine and ephedrine administration during spinal anesthesia for cesarean delivery in high-risk pregnancies, an extensive literature search was conducted through the United States National Library of Medicine using the MEDLINE search engine, Cochrane review, and Google Scholar. The search was limited to articles published in English prior to October 11, 2016. Search terms “ephedrine and phenylephrine,” “preterm and term and spinal hypotension,” “preeclampsia and healthy parturients,” or “multiple and singleton gestation and vasopressor” were used to identify relevant articles. Based on our review, 10 articles that investigated vasopressor use in high-risk human pregnancies were located, and 5 of these specifically compared the effects of phenylephrine and ephedrine in high-risk obstetric patients. Society of Obstetric Anesthesia and Perinatology meeting abstracts for the past 4 years were also searched for studies comparing phenylephrine and ephedrine in high-risk parturients.

## Results and Discussion

### Effect on Uteroplacental Blood Flow

In normal pregnancy, a low-resistance vascular pathway to the intervillous space develops in order to ensure adequate perfusion to meet the needs of the growing fetus and placenta (11–13). Increases in uterine and UA vascular resistance detected on ultrasound surveillance of high-risk pregnancies are important in predicting fetal hypoxia and optimizing fetal outcomes (14–18). Placental perfusion and fetal well-being can also potentially be impacted by both spinal-induced hypotension and the vasopressors used to prevent this hypotension. Despite evidence in favor of phenylephrine in healthy human pregnancies, there is concern regarding potential reductions in uteroplacental blood flow with administration of α_1_-adrenergic agonists based on studies in healthy pregnant ewes and also pregnant ewes with uteroplacental insufficiency (3, 19–21). However, there are several limitations to the application of animal study results to clinical medicine. Different species may have dissimilar adrenergic receptor distribution, affinity to vasopressors, or placental transfer of vasopressors. The human placenta is characterized by a thinner hemomonochorial structure that may allow a greater diffusion of ephedrine compared with the synepitheliochorial placenta of sheep (22). Additionally, animal experiments were performed under isoflurane anesthesia, which might enhance the pulmonary vasodilator response to the β-adrenoceptor agonist (23).

Studies on the effects of ephedrine and phenylephrine administration on human uteroplacental blood flow have been limited to elective cesarean deliveries in uncomplicated pregnancies. Increased resistance in uterine and umbilical artery blood flow is associated with higher velocity indices measured by the systolic/diastolic (S/D) ratio, the pulsatility index (PI), and the resistance index (24). Alahuhta et al. compared the effects of phenylephrine and ephedrine infusions on uteroplacental vascular resistance during spinal anesthesia in healthy parturients and observed a significant increase in the PI of uterine and placental arcuate arteries with phenylephrine administration, though no significant change from baseline with ephedrine (18). Recently, Guo et al. examined the effects of phenylephrine or ephedrine infusion on placental vascular resistance during spinal anesthesia and observed no significant differences in the umbilical artery or uterine artery vascular resistance, though the uterine arterial vascular resistance was elevated from baseline in both study groups (25). No significant differences in fetal acid–base status or clinical outcomes were noted between phenylephrine and ephedrine study groups in either study (18, 25). Ngan Kee et al. investigated the effects of ephedrine and metaraminol on uterine vascular resistance in healthy parturients undergoing elective cesarean section. They found that changes in uterine artery PI were similar between groups; however, the patients receiving ephedrine had lower UA and umbilical venous (UV) pH values (26).

Based on our review, there have not been any studies specifically examining the effects of phenylephrine or ephedrine on uteroplacental blood flow in patients with preeclampsia, uteroplacental insufficiency, or other high-risk conditions. Moreover, an association between prenatal stress and changes in Doppler waveform parameters remain inconclusive due to the methodological limitations of available studies (27). Further investigations are necessary to clarify the effect of vasopressors on uteroplacental blood flow and fetal well-being in these high-risk settings.

### Effect on Fetal Outcome

#### Fetal Outcomes in Uteroplacental Insufficiency

In elective cesarean deliveries, phenylephrine has been associated with higher fetal pH compared with ephedrine (5–10), suggesting that the effects of phenylephrine on uterine blood flow and the fetus are not harmful in healthy pregnant women (28). However, there is less evidence available in cases of unscheduled cesarean deliveries or in the setting of uteroplacental insufficiency. Based on limited available evidence, clinical outcomes of the neonates in the presence of potential uteroplacental insufficiency show no statistically significant differences in UA and UV pH, incidence of fetal acidosis, or Apgar scores between phenylephrine and ephedrine administration (Table 1).

**Table 1:** **Clinical studies comparing the effect of ephedrine and phenylephrine on “fetal outcome” in the setting of uteroplacental hypoperfusion and hypertensive disorders of pregnancy**.

Reference	Study	Inclusion criteria	Group	*N*	Spinal anesthesia medication	BP management	Outcomes			End of study
							UA, UV pH	Neonatal	Maternal	
Ngan Kee et al. (29)	Randomized, double-blinded study	Non-elective cesarean section	No vasopressor	*N* = 56	0.5% hyperbaric bupivacaine 10–12 mg with FEN 15 µg	Bolus if SBP < 100 mmHg	No significant differences between group PE and E	No difference in 1- and 5-min Apgar scores or NICU stay	Similar number of hypotensive episodes	Uterine incision
			PE 100 µg bolus	*N* = 74					Higher incidence of nausea or vomiting in group E	
			E 10 mg bolus	*N* = 74						

Cooper et al. (30)	Retrospective observational study	High-risk cesarean delivery	No vasopressor	*N* = 115	0.5% hyperbaric bupivacaine 11–12.5 mg	Not reported	No significant differences between groups	No difference in the incidence of 5-min Apgar score <7, higher incidence of admissions to neonatal unit in group PE than E	No difference in number of hypotensive episodes	Delivery
			PE infusion started at 33 µg/min	*N* = 97						
			PE 100 µg bolus	*N* = 51						
			E infusion	*N* = 12						
			E 6 mg bolus	*N* = 110						

Mohta et al. (31)	Prospective, randomized, double-blind study	Emergency cesarean section due to fetal compromise	No vasopressor	*N* = 30	0.5% hyperbaric bupivacaine 10–11 mg	Bolus if SBP < 100 mmHg	No significant differences between group PE and E	No significant differences in Apgar scores, number of NICU admissions, or duration of NICU stay	The number of hypotensive episodes were comparable	Delivery
			PE 100 µg bolus	*N* = 53						
			E 8 mg bolus	*N* = 53						

Jain et al. (32)	Prospective, randomized study	Emergency cesarean section due to acute fetal compromise	PE infusion	*N* = 45	0.5% hyperbaric bupivacaine 10 mg with FEN 25 µg	Bolus if SBP < 90% of baseline	No significant differences between group PE and E	No difference in number of low 1-min Apgar scores	Mean SBP was comparable	Delivery
			30 µg/min + PE bolus 50 µg							
			E infusion	*N* = 45		Infusion rate reduced if SBP 110–120%		No warrant further observation in pediatric care unit after 24-h F/U	Higher incidence of nausea or vomiting in group E	
			2.5 mg/min + E bolus 4 mg			Stopped if SBP > 120%				

Ituk et al. (37)	Retrospective observational study	Preeclampsia undergoing cesarean delivery	PE 100 µg bolus	*N* = 57	0.5% hyperbaric bupivacaine 12 mg with FEN 25 µg + MO 250 µg	Not reported	No differences in neonatal UA pH	No difference	Not reported	Delivery
			E 5 mg bolus	*N* = 89				In 1- and 5-min Apgar score		

Higgins et al. (38)	Prospective, randomized study	Preeclampsia undergoing cesarean delivery	PE infusion 100 μg/min	*N* = 54	Not reported	Titrated to keep SBP > 80% of baseline but not >160 mmHg	No significant differences in median UA pH	No difference in 1- or 5-min Apgar score or NICU admission	No difference in maternal SBP	Delivery
			E infusion 8 mg/min	*N* = 54						

*BP, blood pressure; SBP, systolic blood pressure; PE, phenylephrine; E, ephedrine; UA pH, umbilical artery pH; UV pH, umbilical venous pH; FEN, fentanyl; MO, morphine; F/U, follow-up; NICU, neonatal intensive care unit*.

A prospective study published by Ngan Kee et al. (29) enrolled 204 patients who were randomized to receive intermittent boluses of either phenylephrine (100 μg) or ephedrine (10 mg) to treat episodes of spinal-induced hypotension (defined as systolic BP ≤100 mmHg) during non-elective cesarean deliveries. Neonatal Apgar scores, UA pH, UV pH, and base excess values were not significantly different between ephedrine and phenylephrine groups, though higher UA and UV lactate concentrations and greater incidence of nausea and vomiting were observed in patients receiving ephedrine (29). It is important to note that this study included patients in which cesarean deliveries were booked on the day of surgery, including patients in the labor ward who eventually underwent cesarean delivery. Potential fetal compromise was noted in 48 patients in this study. The subanalysis of these cases noted similar UA and UV blood gas parameters and lactate concentrations in phenylephrine and ephedrine groups, with the exception of a lower UA PO_2_ in the phenylephrine group. It is important to note that patients with preexisting hypertension or pregnancy-induced hypertension were excluded from this investigation (29).

In 2010, Cooper et al. presented a retrospective observational study in patients with increased risk of fetal compromise who underwent cesarean delivery under spinal anesthesia (30). Prophylactic infusions of phenylephrine (33 μg/min) or ephedrine and intermittent boluses of either ephedrine (6 mg) or phenylephrine (100 μg) were all utilized as the primary means of preventing or treating spinal-induced hypotension. Similar neonatal Apgar scores and UA pH values were found in patients receiving ephedrine, phenylephrine, or no vasopressors (30). Notably, the incidence of pregnancy-induced hypertension was significantly greater among patients not requiring vasopressors. Multiple regression analysis noted the only variable associated with altered UA pH was a non-reassuring fetal heart rate (HR) tracing prior to cesarean delivery. The authors noted that increased incidence of prematurity and shorter spinal-to-delivery intervals due to the urgent nature of these cases might affect total dose of ephedrine given before delivery. In this study, the ephedrine dose was much lower than ephedrine dose used in low-risk cesarean deliveries (12 vs. 52 mg), thereby reducing potential adverse metabolic effects in the fetus (30). Ngan Kee et al. (29) administered similar low doses of ephedrine (median 10 mg) in their prospective study of non-elective cesarean deliveries compared to the total dose of ephedrine (median 54 mg) reported in their study of elective cesarean deliveries in low-risk patients (8).

A recent study by Mohta et al. in patients undergoing cesarean deliveries due to potential fetal compromise reported data from 106 patients randomized to receive either ephedrine (8 mg) or phenylephrine (100 mg) boluses to treat episodes of spinal-induced hypotension (systolic BP ≤100 mmHg) (31). The number of vasopressor boluses and hypotensive episodes were similar between phenylephrine and ephedrine groups. No statistically significant intergroup differences were noted in UA and UV pH, incidence of fetal acidosis, or Apgar scores. The spinal-to-delivery interval in these urgent cesarean deliveries was relatively short (mean 9 min), and the authors suggest that this may have contributed to the reduced ephedrine requirement and decreased fetal drug exposure (31). Patients with preeclampsia were excluded from this investigation (31).

Another recent investigation by Jain et al. prospectively compared prophylactic ephedrine infusion (2.5 mg/min) to phenylephrine infusion (30 μg/min) to manage spinal-induced hypotension in 90 patients undergoing cesarean delivery due to signs of acute fetal compromise in the intrapartum period (32). The authors reported no differences in fetal acidosis with either prophylactic phenylephrine or ephedrine infusion and no adverse neonatal outcomes during the period of observation in the study (32).

#### Fetal Outcomes in Hypertensive Disorders of Pregnancy

Hypertensive disorders in pregnancy are associated with maternal morbidity and mortality, accounting for 9.4% of pregnancy-related deaths in the United States during 2006–2010 (33). Inadequate trophoblast invasion, leading to incomplete remodeling of the uterine spiral arteries, is considered to be a primary cause of the placental ischemia (11–13). The resulting elevated vascular resistance may account for the differences in reactivity of vessels to vasoconstrictor drugs (34). There is concern that administering vasopressors may impair uteroplacental blood flow in preeclamptic patients given the increased responsiveness to vasomotor stimuli.

The incidence of spinal-induced hypotension and the need for vasopressor treatment has been noted to be reduced in patients with preeclampsia in comparison to both healthy term parturients (35) and preterm cesarean deliveries (36). The authors of these investigations conclude that preeclampsia-associated factors likely account for the lower incidence of hypotension (35, 36). Average ephedrine doses of 6–10 mg were administered in preeclamptic patients without reports of adverse maternal or fetal events in these investigations (35, 36). Similarly, a retrospective study by Cooper et al. noted that the patients were less likely to require vasopressor support for hypotension in the setting of pregnancy-induced hypertension (30).

Few studies have directly compared the use of ephedrine and phenylephrine for spinal-induced hypotension in preeclamptic patients. Cooper et al. reported that non-reassuring fetal HR tracing was the only variable associated with lower UA pH on multiple regression analysis, while the use of ephedrine, phenylephrine, or the presence of pregnancy-induced hypertension were not associated with alterations in UA pH (30). Ituk et al. also performed a retrospective study comparing phenylephrine with ephedrine for managing hypotension after spinal anesthesia in preeclamptic patients and reported no difference in UA pH between the two study groups (37). However, the ephedrine group was characterized by significantly lower gestational age at delivery compared to the phenylephrine group (mean 32 vs. 36 weeks gestation), which may have contributed to reduced incidence of hypotension and fewer doses of vasopressors due to reduced aortocaval compression by the smaller fetus (37). Similar to Cooper et al. (30), this study found that non-reassuring fetal heart tracing prior to delivery was the only factor significantly associated with lower UA pH (37). An abstract presented at the Society of Obstetric Anesthesia and Perinatology (SOAP) 2015 conference described a prospective study comparing phenylephrine infusion (100 μg/min) or ephedrine infusion (8 mg/min) for prevention of spinal-induced hypotension in preeclamptic patients. They observed similar UA pH values and incidence of fetal acidosis (UA pH < 7.20) in phenylephrine and ephedrine study groups and concluded that both vasopressors appear to be safe in preeclampsia (38) (Table 1).

Based on the limited data available, it appears that both ephedrine and phenylephrine are suitable options for managing spinal-induced hypotension in patients with hypertensive disorders of pregnancy. In women with preeclampsia, there are no apparent differences between phenylephrine and ephedrine with regard to UA or UV pH, and neonatal outcomes (30, 37, 38). The reduced incidence of spinal-induced hypotension in preeclampsia (35, 36) and correspondingly lower doses of vasopressors may account for these observations.

### Effect on Maternal Outcome

In the studies reviewed, a variety of phenylephrine and ephedrine dosing regimens were used for either prevention of spinal-induced hypotension or to treat hypotension in high-risk obstetric patients, making it difficult to draw conclusions. Based on the limited available evidence, phenylephrine and ephedrine appear to be similarly effective in preventing and treating spinal-induced hypotension in parturients with potential uteroplacental insufficiency (29–32) or hypertensive disorders of pregnancy (30, 37, 38). These findings diverge from studies in healthy parturients, in which phenylephrine has been observed to be more effective at maintaining maternal blood pressure (7, 8) and preventing IONV (5, 7, 8) compared to ephedrine. Compared to healthy pregnancies, there appears to be reduced incidence of hypotension and lower vasopressor requirements in patients with uteroplacental insufficiency or hypertensive disorders of pregnancy (29–32, 37), which may be due to a combination of increased incidence of prematurity, shorter incision-to-delivery intervals in urgent circumstances, or preeclampsia-associated factors.

During spinal anesthesia, preeclampsia patients maintain a high vascular tone and typically display a limited decrease in mean arterial pressure. Preeclampsia-induced endothelial dysfunction leads to increases in endothelin and thromboxane production, decreased vasodilator synthesis, and sensitizes the vasculature to vasoconstrictors (39). Previous studies indicated that spinal anesthesia-induced hypotension was less frequent and less severe in preeclamptic patients when compared to normotensive parturients, and minimal doses of vasopressors are typically required to restore maternal blood pressure to baseline (35, 36, 40) (Table 2). Similarly, Ituk et al. recently conducted a retrospective comparison of phenylephrine and ephedrine for management of spinal-induced hypotension in preeclamptic patients and observed that approximately 50% of patients did not experience hypotension requiring vasopressor treatment following spinal anesthesia (37).

**Table 2:** **Vasopressor use in high risk pregnancy compared to normal pregnancy undergoing Cesarean delivery**.

Reference	Participants	*N*	IV preload	Neuraxial anesthesia medication	Upper sensory level	Non-invasive BP monitoring	Definitions of hypotension	Intervention	Outcomes		
									Hemodynamics	IV fluid	Vasopressor
Aya et al. (35)	Severe preeclamptics	*N* = 30	1,500–2,000 mL of lactated Ringer’s solution over 20 min	SAB with 0.5% hyperbaric bupivacaine 8–12 mg + SUF 3–5 µg + MO 100 µg	T4 (T3–T5)	2-min intervals from SAB for 30 min and then at 5-min intervals until the end of the surgery	SBP < 100 mmHg in healthy parturients or 30% decrease in mean BP (in both groups)	E 6 mg bolus repeated every 2 min	Lower incidence of hypotension in preeclamptic group	Smaller preload volume in preeclamptic group	Less ephedrine requirement in preeclamptic group
	Healthy parturients	*N* = 30			T4 (T2–T5)				Incidence of heart rate (HR) changes <20% was similar		

Aya et al. (36)	Severe preeclamptics	*N* = 65	1,500–2,000 mL of lactated Ringer’s solution over 20 min	SAB with 0.5% hyperbaric bupivacaine 8–12 mg + SUF 3–5 µg + MO 100 µg	T4 (T3–T5)	2-min intervals from SAB for 30 min and then at 5-min intervals until the end of the surgery	SBP < 100 mmHg in healthy parturients or 30% decrease in mean BP (in both groups)	E 6 mg bolus repeated every 2 min	Lower incidence of hypotension in preeclamptic group	No significant differences between groups	Less ephedrine requirement in preeclamptic group
	Preterm pregnancies	*N* = 71			T4 (T2–T5)				Incidence of HR changes <20% was similar		

Nikooseresht et al. (40)	Severe preeclamptics	*N* = 43	10 mL/kg of Ringer’s lactate solution over 15–20 min	SAB with 0.5% hyperbaric bupivacaine 10 mg + SUF 2.5–3 µg	T4 (T2–T5)	2-min intervals from SAB for 15 min and then at 5-min intervals until the end of the surgery	SBP < 100 mmHg in healthy parturients or 25% decrease in mean BP (in both groups)	E 5 mg bolus	Lower incidence of hypotension in preeclamptic group	Smaller volumes of intravenous fluids in preeclamptic group	Less ephedrine requirement in preeclamptic group
	Healthy parturients	*N* = 37			T4 (T2–T5)						

James et al. (41)	Term pregnancy	*N* = 25	15 mL/kg of crystalloid solution	SAB with 0.5% hyperbaric bupivacaine 11.25 mg + EPI 2% lignocaine with ADR	T3	Not reported	SBP < 70% of the baseline	Not reported	Lower incidence of hypotension in preterm group	Not reported	Not reported
	Preterm pregnancy	*N* = 25			T4						

Ngan Kee et al. (43)	Multiple gestation pregnancy	*N* = 40	20 mL/kg lactated Ringer’s solution over 15–20 min	SAB with 0.5% hyperbaric bupivacaine 10 mg + FEN 15 µg	T4 (T2–T5)	1-min intervals from SAB until uterine incision	SBP < 80% of the baseline	MET infusion 0.25 mg/min + MET bolus 0.5 mg	No differences in the incidences of hypotension	Not reported	No differences in total dose of MET
					T4 (T3–T4)						
	Singleton pregnancy	*N* = 60									

*BP, blood pressure; SBP, systolic blood pressure; MAP, mean arterial pressure; SAB, subarachnoid block; EPI, epidural block; SUF, sufentanil; MO, morphine; FEN, fentanyl; ADR, adrenaline (1:2,000,000); PE, phenylephrine; E, ephedrine; MET, metaraminol*.

Preterm women have less aortocaval compression due to a smaller uterine mass, which has been observed to decrease the incidence of hypotension and requires smaller doses of vasopressors than those in term pregnancy (41). However, the frequency and magnitude of spinal hypotension in preeclampsia patients has been observed to be less than in women with preterm pregnancies (36). The risk of hypotension among the preeclamptic group was almost two times lower than that in the preterm group (relative risk = 0.603; 95% confidence interval, 0.362–1.003; *P* = 0.044), and the ephedrine requirement to restore blood pressure to baseline level was less than in the preterm group (9.8 ± 4.6 vs. 15.8 ± 6.2 mg, respectively, *P* = 0.031) (36).

On the other hand, multiple gestations do not appear to be a risk factor for developing hypotension after regional anesthesia for cesarean deliveries, despite having a greater uterine mass (42). Ngan Kee et al. (43) conducted a prospective study comparing vasopressor requirements in twin gestations vs. singletons. There were no differences in the incidences of hypotension, nausea, vomiting, or vasopressor dosage between study groups (Table 2).

The maternal HR is significantly higher after ephedrine administration (29, 31, 32), while the incidence of maternal bradycardia is significantly greater after phenylephrine administration (29, 31, 32), though these differences do not appear to significantly impact clinical outcomes in high-risk obstetric patients. As a selective α_1_-adrenergic receptor agonist, phenylephrine-induced increases in blood pressure activate the baroreceptor reflex and cause bradycardia. However, these bradycardic events can be minimized or prevented by careful bolus dosing of phenylephrine, or minimizing the infusion rate. Furthermore, with these measures in place, no detrimental effects on maternal and neonatal outcome have been observed in normal (44) or in high-risk parturients (29, 31, 32).

## Conclusion

The management of hypotension during cesarean delivery under spinal anesthesia remains challenging. The administration of vasopressors is often necessary, despite other measures such as intravenous crystalloid infusions. Phenylephrine is currently the vasopressor of choice for preventing or treating spinal-induced hypotension in many practices, as many studies in elective cesarean deliveries have demonstrated phenylephrine to be associated with more favorable fetal acid–base status and greater effectiveness in preventing hypotension and IONV. However, phenylephrine use in the presence of preexisting fetal compromise continues to be controversial due to concern of potential uterine blood flow reduction.

Based on limited available evidence, it appears that phenylephrine and ephedrine are similarly safe and effective, and both may be used to prevent or treat spinal-induced hypotension in the setting of potential uteroplacental insufficiency and preeclampsia. The current literature does not show any statistically significant differences in terms of maternal and neonatal outcomes between phenylephrine and ephedrine in these high-risk patients. Further randomized double-blind studies are required to further clarify the safety and efficacy and determine effective vasopressor dosing regimens in high-risk obstetric patients.

## Author Contributions

SD and BH conducted a review of the literature. SD, MS, EB, BH, DS, and JC prepared the body of the manuscript. JC critically reviewed the publication. All the authors endorsed the final form of the manuscript.

## Conflict of Interest Statement

The authors declare that the research was conducted in the absence of any commercial or financial relationships that could be construed as a potential conflict of interest.
